# Polymorphisms in lncRNA *CCAT1* on the susceptibility of lung cancer in a Chinese northeast population: A case–control study

**DOI:** 10.1002/cam4.4902

**Published:** 2022-06-01

**Authors:** Yangtao Ji, Yue Yang, Zhihua Yin

**Affiliations:** ^1^ Department of Laboratory Medicine, National Clinical Research Center for Laboratory Medicine The First Affiliated Hospital of China Medical University Shenyang Liaoning People's Republic of China; ^2^ Department of Laboratory Medicine The First Affiliated Hospital of China Medical University Shenyang Liaoning People's Republic of China; ^3^ Department of Epidemiology School of Public Health, China Medical University Shenyang Liaoning People's Republic of China

**Keywords:** *CCAT1*, lncRNA, lung cancer, rs6983267, SNP

## Abstract

**Object:**

To explore the association of rs1948915, rs7013433 in long noncoding RNA (lncRNA) *CCAT1* and rs6983267 in *MYC* enhancer region with the risk of lung cancer in a Chinese northeast population, a case–control study was conducted.

**Methods:**

The hospital‐based case–control study contained 669 lung cancer patients and 697 healthy controls. Taqman® Probe allele resolution was used for genotyping. The differences between the case–control groups were analyzed using Student *t*‐test and chi‐square test. Logistic regression analysis was used to assess the relationship between the genotypes and the risk of lung cancer. Cross‐generation analysis was used to explore the relationship between gene–environment interaction and lung cancer.

**Results:**

There was no association between the three selected single‐nucleotide polymorphisms (SNPs) and the susceptibility of lung cancer. Rs1948915 CT was correlated with lung adenocarcinoma. In female stratification, rs1948915 CT/CC was associated with a decreased susceptibility of lung cancer significantly. Additionally, the additive and multiplicative interaction models showed that there was no interaction between the three selected SNPs and smoking status in lung cancer.

**Conclusions:**

There may be an association between lung adenocarcinoma and rs1948915 polymorphism in the Chinese northeast population, while rs7013433 and rs6983267 might have no association. There was no interaction between the three selected SNPs and smoking status.

## Graphical abstract

There may be an association between lung adenocarcinoma and rs1948915 in lncRNA *CCAT1* in the Chinese northeast population, while rs7013433 in lncRNA *CCAT1* and rs6983267 in *MYC* enhancer region might have no association. There was no interaction between the three selected SNPs and smoking status.

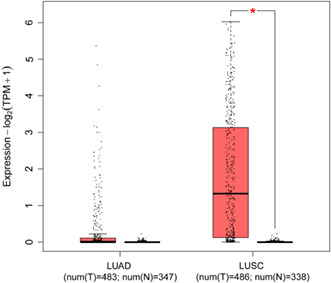

## 1 INTRODUCTION

Cancer is one of the most serious diseases nowadays, which cannot be ignored. According to the International Agency for Research on Cancer (IARC), 18.1 million new cases and 9.6 million cancer deaths were estimated worldwide in 2018.^1^ By 2030, the global burden of cancer will rise to around 22.2 million new cases and 13.2 million deaths, which is disturbing.^2^ Notably, there were 2.1 million people newly diagnosed with lung cancer and 1.8 million people died of it.^1^ What is more, the 5‐year survival rate of lung cancer is as low as 19% behind only pancreatic cancer, suggesting that increasing attention should be given to lung cancer.^3^

Accumulating researches have indicated that the occurrence of lung cancer is an intricate process, which is affected by a variety of factors, including genetic factors, environmental factors, and their interaction.^4^, ^5^ With the burgeoning growth of genome‐wide association studies (GWAS), a large number of studies have emerged on the relationship between long noncoding RNA (lncRNA) single‐nucleotide polymorphisms (SNPs) and cancer susceptibility, as well as a greater focus on genetic risk factor studies. LncRNA, over 200 nucleotides, is a kind of noncoding RNA that has no protein‐coding function.^6^, ^7^ According to the function in tumors, lncRNAs can be divided into tumor‐promoting lncRNAs and tumor‐suppressive lncRNAs. As gene regulators, lncRNAs may play an imperative role in trans‐, cis‐, and post‐transcriptional gene regulation through complex mechanisms in oncogenic paths.^8^, ^9^, ^10^, ^11^ LncRNA polymorphisms might regulate their functions and expressions, affecting individual's cancer susceptibility.^12^, ^13^, ^14^ That is to say, polymorphisms in functional lncRNAs, just like SNPs of protein‐coding genes, can also promote the development of cancer.^15^, ^16^

*CCAT1* (colon cancer‐associated transcript 1), also termed as *LOC100507056* or *CARLo‐5* (cancer‐associated region long noncoding RNA), is a 2682 nucleotide‐long lncRNA near *c‐Myc* on chromosome 8q24.21, a famous transcription factor.^17^, ^18^, ^19^, ^20^, ^21^ In 2012, Nissan et al. measured the high specific expression of *CCAT1* in colorectal cancer (CRC) for the first time, and was once considered to be a specific expression of lncRNA in CRC, reporting that the average expression level of *CCAT1* in colon cancer tissues was 235 times uncommonly higher than the counterpart in normal colon mucosa tissues.^19^ However, emerging studies have recognized that the overexpression of lncRNA *CCAT1* was determined in many types of cancer, like gastric carcinoma (GC)^20^ and hepatocellular carcinoma (HCC),^22^ etc. besides CRC.

The expression of *miR490* can be regulated by *CCAT1* in gastric cancer, while *miR490* can also inhibit *CCAT1* expression, and they are negatively correlated, whose high expression after transcription can decrease the expression of *CCAT1* and significantly restrain the metastasis of gastric cancer.^23^ Upregulation of *CCAT1* expression is directly related to *c‐Myc* in the E‐box (enhancer box) element of its gene promoter region. If the E‐box element mutates, *c‐Myc* will not promote *CCAT1* expression.^24^, ^25^, ^26^ Xiang et al. showed *CCAT1* promoted long‐range chromatin looping and regulated the process of *MYC* transcription. The absence of *CCAT1* decreased long‐range interaction between its enhancers and the *c‐MYC* promoter.^17^ LncRNA *CCAT1* is closely correlated to *c‐MYC* transcription and cell growth in a variety of cancer types.^27^, ^28^ Zhao et al. have reported that *CCAT1* expression is closely regulated by carcinogenic SNP rs6983267 of the *MYC* enhancer region, correlated with endometrial carcinoma.^29^

Various studies have shown an association between lncRNA CCAT1 polymorphisms and cancer susceptibility. Previous studies analyzed European patients with multiple myeloma by GWAS and found that lncRNA *CCAT1* rs1948915 polymorphism was closely related to multiple myeloma.^30^, ^31^ Li et al. concluded that lncRNA *CCAT1* rs7013433 polymorphism was tightly connected with advanced stage of colorectal cancer in the population of Fujian and Zhejiang provinces, China, through a case–control study.^32^ Park et al. found that SNP rs69832627 was connected with the susceptibility of lung cancer in smoking stratification through a case–control study.^33^ Nevertheless, Zhang et al. proposed that subjects with GG homozygous genotype increased the susceptibility of developing lung cancer than individuals carrying TT homozygous genotype in the population of China. Additionally, there was a more significant difference in non‐smokers in smoking stratification.^34^ The conclusions drawn above are prominently inconsistent and need to be verified.

SNP rs6983267 is located on lncRNA *CCAT2*. Many studies have shown that the high expression of *CCAT1* and *CCAT2* is significantly related to the poor prognosis of CRC patients, and has a strongly association with MYC enhancer.^35^ And these two lncRNA independently, or in combination, can be used as an important biomarker for the prognosis of CRC.^35^, ^36^ Therefore, rs1948915 and rs7013433 in *CCAT1* and rs6983267 in *MYC* enhancer region were selected for this study. Considering the significant parts of *CCAT1* in the development of cancer and the unclear effects of *CCAT1* in lung cancer, we implemented a case–control study to analyze the relationship of the polymorphisms rs1948915, rs7013433 in lncRNA *CCAT1* and rs6983267 in the *MYC* enhancer region with lung cancer susceptibility in the northeast of China. Consequently, we explored the interaction of the selected SNPs and smoking exposure status with the risk of lung cancer, which was necessary to probe cancer etiology and decrease environmental‐related risk factors for cancer prevention.

## 2 MATERIALS AND METHODS

### 2.1 GEPIA2 dataset

GEPIA2 (Gene Expression Profiling Interactive Analysis 2) is an online bioinformatics tool for analyzing the RNA sequencing expression data of 9736 tumors and 8587 normal samples from The Cancer Genome Atlas (TCGA) and the Genotype‐Tissue Expression (GTEx) projects, using a standard processing pipeline (http://gepia2.cancer‐pku.cn/#analysis). To mine the expression and prognosis, GEPIA2 provides customizable tumor/normal differential expression analysis, patient survival analysis and so on.^37^

### 2.2 Study subjects

We performed a hospital‐based case–control study, in Shenyang City, northeast of China, where there were 669 cases and 697 healthy controls. These patients were confirmed as lung cancer (from January 2011 to December 2013) at The First Affiliated Hospital of China Medical University, The Fourth Affiliated Hospital of China Medical University and General Hospital of the Northern War Zone of the Chinese People's Liberation Army. During the same period, we selected the corresponding controls from physical examination in the same hospital. The included criteria of cases were: (1) patients newly diagnosed by two expert pathologists without metastatic cancer or any previous cancer, (2) no therapy (both radiotherapy and chemotherapy), and (3) willing and capable to have an interview. The included criteria of healthy controls were without a history of any cancer or other diseases of lung. Importantly, all the participants are no blood relationship with each other and Chinese Han population. All anticipants were sure not to accept blood transfusion in the past 6 months. We got approval from the Ethics Committee of China Medical University, and informed consent was signed by each subject. After an interview, 10 ml of peripheral venous blood was donated by each subject as specimen for SNP genotyping. Additionally, if a subject smoked under 100 cigarettes in the past, he or she was determined as a non‐smoker; if not, the subject was a smoker.

### 2.3 SNP selection and genotyping

We selected the tagSNPs of *CCAT1* by the pairwise option of the Haploview 4.2 software (setting *r* ^2^ ≥ 0.8, minor allele frequency > 0.05), using the data of Han Chinese from the 1000 Genome Projects. Then, we combined the domestic and foreign studies. Finally, we select rs1948915, rs7013433 in lncRNA *CCAT1* and rs6983267 in the *MYC* enhancer region. The IDs of the test primers in order are C_3052970_10, C_1523520_20, and C_29086771_20. The minor allele frequencies (MAF) of the selected SNPs are totally more than 5% in the population of China. Genomic DNA samples were isolated from venous blood by phenol‐chloroform method. Next, an Applied Biosystems 7500 Real‐Time PCR System (Foster City, CA) was used with Taqman® allelic discrimination for SNP genotyping with primer probe set. There were appropriate positive, negative, and blank controls contained in each run. Over 10% of samples twice were chosen twice randomly and tested for quality control by two persons, and the two results were in concordance with each other completely.

### 2.4 Statistical analysis

We tested the differences of demographic variables between case group and control group with chi‐squared test and Student's *t*‐test. It was confirmed whether the selected SNPs were under the Hardy–Weinberg equilibrium (HWE) in the control population by the goodness‐of‐fit chi‐squared test. We got the ORs and their 95% confidence intervals (CIs) by unconditional logistic regression analysis to estimate the associations of the selected SNPs with the susceptibility of lung cancer. The relationship of the interaction of polymorphisms of the selected SNPs and smoking status with lung cancer was evaluated by logistic regression models (multiplicative interaction) and crossover analysis (additive interaction). We regarded those with both the protective genotype and no smoking exposure as the reference group in our analysis. By logistic regression models, multiplicative interaction was included in the models. All the statistical analyses were conducted by SPSS software (Version 20.0; IBM SPSS, Inc., Chicago, IL, USA). More importantly, we defined statistical significance as *p* < 0.05 for two sides.

## 3 RESULTS

### 3.1 Expression analysis of *CCAT1* in lung adenocarcinoma (LUAD) and lung squamous cell carcinoma (LUSC) tissues

**FIGURE 1:**
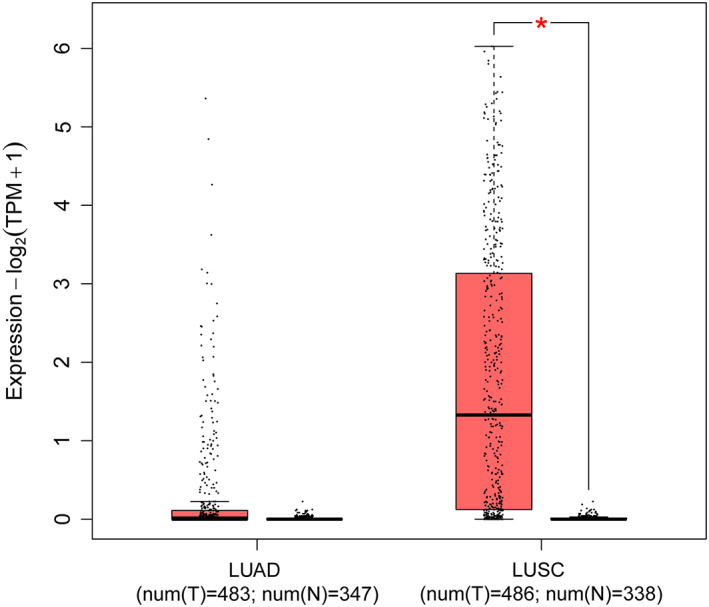
Expression of lncRNA *CCAT1* in LUAD and LUSC tissues samples from TCGA database (LUAD = 483 normal = 347, *p* > 0.05; LUSC = 486; normal = 338, *p* < 0.05.

**FIGURE 2:**
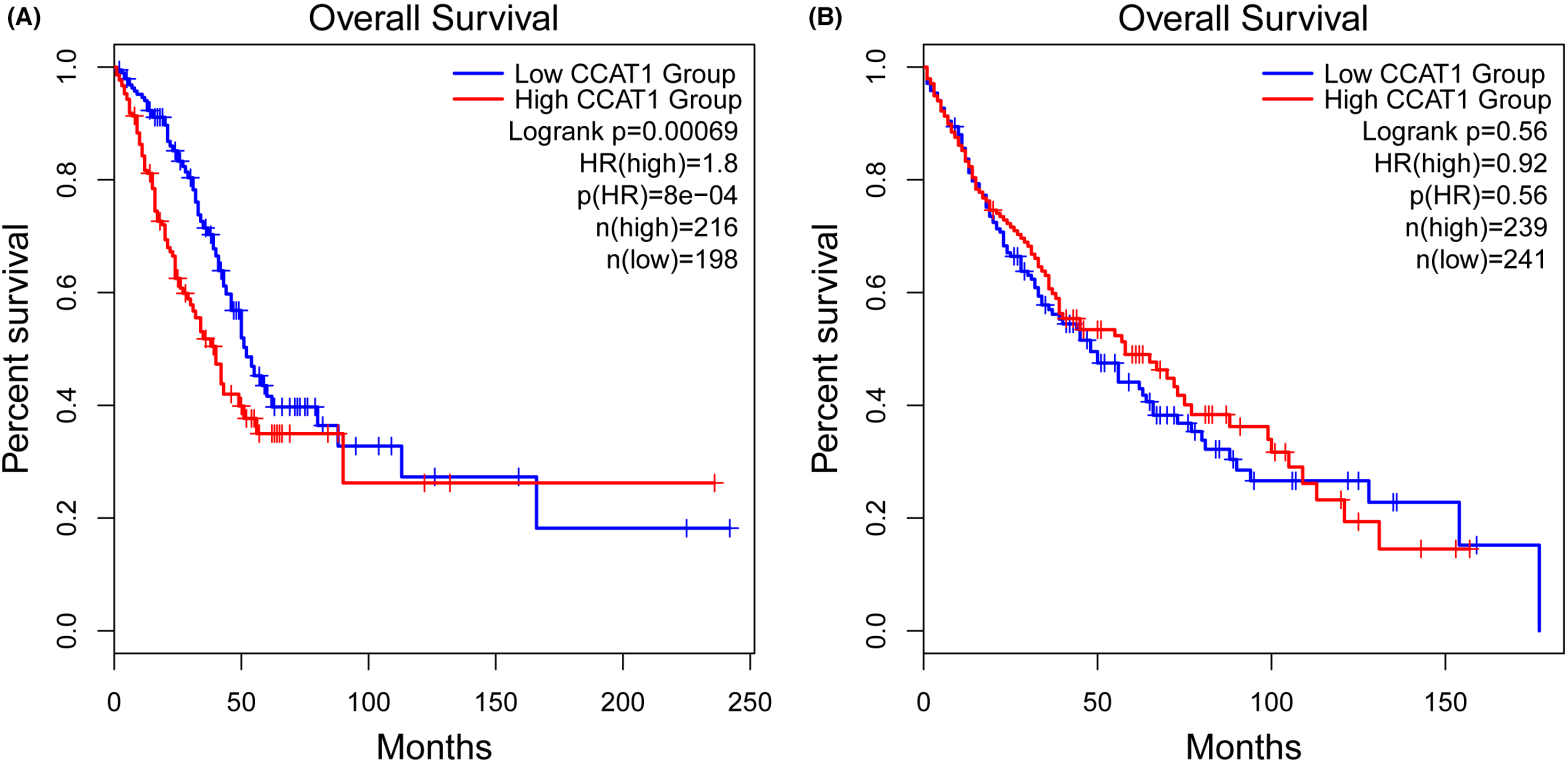
(A) Validation of the prognostic lncRNA *CCAT1* in GEPIA2 from TCGA database (A: Kaplan–Meier curve of the risk score for the Overall Survival of LUAD patients, *p* < 0.05). (B) Validation of the prognostic lncRNA *CCAT1* in GEPIA2 from TCGA database (B: Kaplan–Meier curve of the risk score for the Overall Survival of LUSC patients, *p* > 0.05).

**FIGURE 3:**
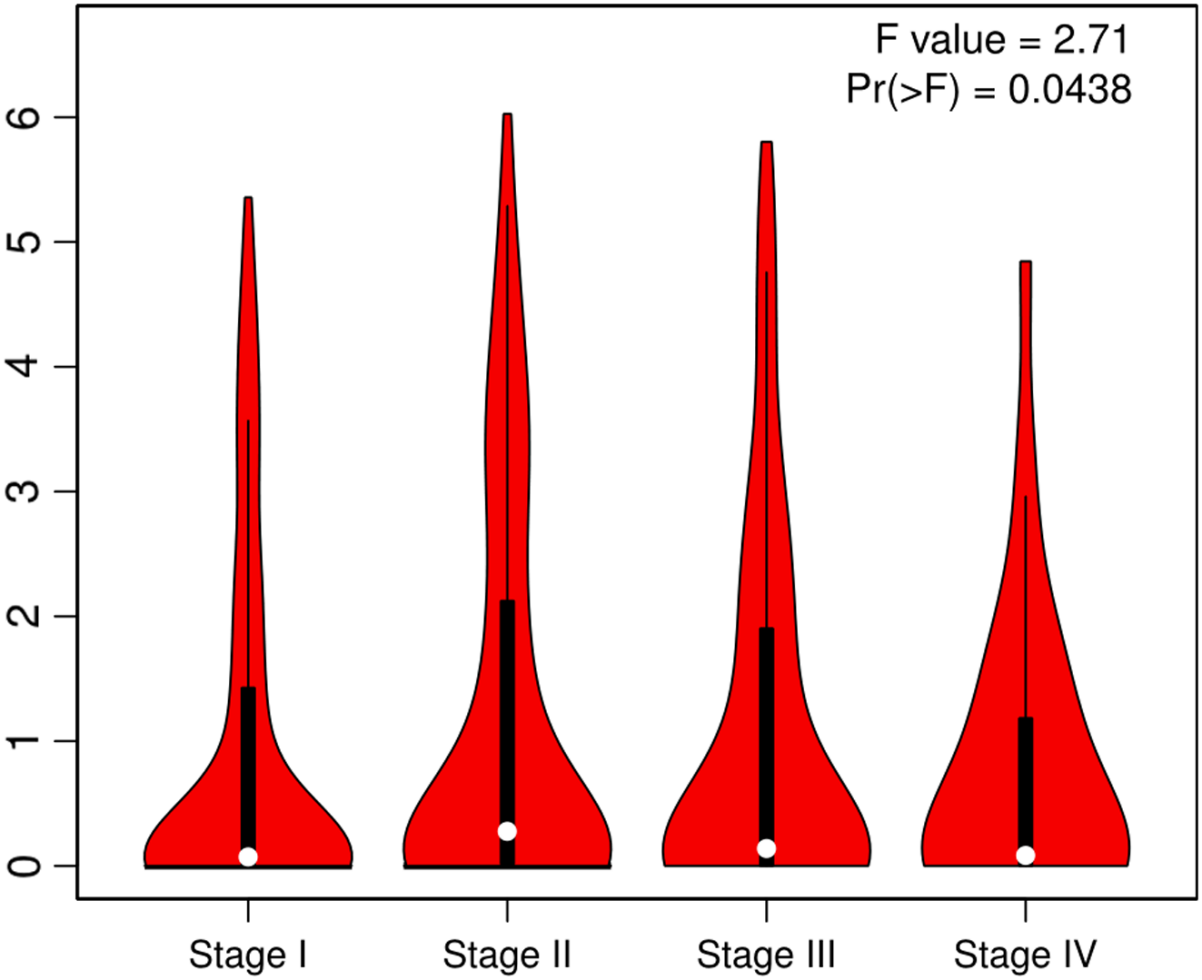
The significant differences in gene expression between different pathological stages in LUAD and LUSC patients, *p* < 0.05.

The *CCAT1* expression of LUSC tissues was significantly higher than that in normal tissues through GEPIA2, including 486 LUSC tissues and 338 normal tissues, while it was no significance between 483 LUAD tissues and 347 normal tissues in Figure 1. The overall survival time showed positive results between clinical samples and the expression level of lncRNA *CCAT1* in LUAD patients (Figure 2 *p* < 0.01), but there was no statistical significance of expression level in LUSC patients (Figure 2B, *p* > 0.05). We also generated expression violin maps based on the patient's stage of pathology. The results showed statistically significant differences in gene expression between different pathological stages in LUAD and LUSC patients (Figure 3, *p* < 0.05).

### 3.2 Baseline characteristics

This epidemiologic study recruited a total of 1366 participants including 669 patients with lung cancer and 697 healthy controls, whose demographic characteristics are depicted in Table 1. In the case group, there were 541 non‐small cell lung cancer (NSCLC) patients, 107 small cell lung cancer (SCLC) patients, and 21 other types. Among the NSCLC patients, 282 were adenocarcinoma cases, 248 were squamous cell carcinoma cases, 10 were large cell carcinoma patients, and 1 mixed cancer patient. There was no statistical significance in the distribution of gender between the cases and controls (*p* = 0.068). Under our anticipation, the smoking exposure rate was 51.9% in the patients, whereas it was 31% in the control group, showing that the smoking exposure was an evident risk factor to lung cancer (*p* < 0.001). However, the Student‐*t* revealed that the age distribution between the two groups was statistically significant, with being 60.51 ± 11.12 and 56.26 ± 14.97 in the cases and controls separately (*p* < 0.001). Therefore, all further statistical analyses were adjusted by gender, age, and smoking status to eliminate the potential confounders. The genotype frequencies of rs1948915, rs7013433, and rs6983267 in the control group were under the Hardy–Weinberg equilibrium (*χ* ^2^ = 1.049, *p* = 0.306 for rs1948915; χ^2^ = 0.0006, *p* = 0.980 for rs7013433; χ^2^ = 0.419, *p* = 0.518 for rs6983267), indicating that the subjects selected were a good representative sample from the general population.

**TABLE 1:** Demographics of cases and controls

Variable	Case (%)	Control (%)	*p*
Number	669	697	
Age, year (Mean ± SD)	60.51 ± 11.12	56.26 ± 14.97	**<0.001**
Age year			
>58	383 **(**57.2**)**	337 **(**48.4**)**	**<0.001**
≤58	286 **(**42.8**)**	360 **(**51.6**)**	
Gender			
Male	437 **(**65.3**)**	422 **(**60.5**)**	0.068
Female	232 **(**34.7**)**	275 **(**39.5**)**	
Smoking status			
Ever	347 **(**51.9**)**	216 **(**31.0**)**	**<0.001**
Never	322 **(**48.1**)**	481 **(**69.0**)**	
Pathological type			
Adenocarcinoma	282		
Squamous cell carcinoma	248		
Small cell lung cancer	107		
Other	32		

### 3.3 Genotype distribution and lung cancer susceptibility

It was summarized that the relationship of the genotype results of the three SNPs with the susceptibility to lung cancer and NSCLC in Table 2. There was no statistical significance in all models for rs1948915 polymorphism (CT vs. TT: OR = 0.782, 95% CI = 0.600–1.020, *p* = 0.069; CC vs. TT: OR = 0.877, 95% CI = 0.641–1.199, *p* = 0.410; CC + CT vs. TT: OR = 0.811, 95% CI = 0.632–1.041, *p* = 0.101; CC vs. CT + TT: OR = 1.030, 95% CI = 0.794–1.336, *p* = 0.826, adjusted by age, gender, and smoking status). Rs7013433 genetic variant was not associated with lung cancer (AT vs. TT: OR = 0.887, 95% CI = 0.688–1.143, *p* = 0.354; AA vs. TT: OR = 0.961, 95% CI = 0.702–1.314, *p* = 0.802; AA + AT vs. TT: OR = 0.908, 95% CI = 0.715–1.154, *p* = 0.43; AA vs. AT + TT: OR = 1.034, 95% CI = 0.789–1.357, *p* = 0.806, adjusted by age, gender, and smoking status). The result of rs6983267 polymorphism was also negative in all models (GT vs. TT: OR = 0.972, 95% CI = 0.761–1.240, *p* = 0.817; GG vs. TT: OR = 0.972, 95% CI = 0.761–1.240, *p* = 0.872; GG + GT vs. TT: OR = 0.972, 95% CI = 0.771–1.225, *p* = 0.809; GG vs. GT + TT: OR = 0.989, 95% CI = 0.73–1.341, *p* = 0.945, adjusted by age, gender, and smoking status). Furthermore, these above results were similar with counterparts in NSCLC and lung squamous cell carcinoma subgroups (shown in Tables 2 and 3). The similar result appeared in stratification analysis of smoking exposure (shown in Table S1). Among the smoking population, there is no association of polymorphisms of the selected SNPs with lung cancer susceptibility in the whole of models. However, rs1948915 CT was correlated with lung adenocarcinoma, compared with TT genotype (CT vs. TT: OR = 0.688, 95% CI = 0.491–0.963, *p* = 0.029). Tables 4 and 6 presented stratification analysis results of gender and age, respectively. In female population, rs1948915 CT/CC was related to significantly decreased risk of lung cancer (CT vs. TT: OR = 0.610, 95% CI = 0.396–0.941, *p* = 0.025; CC vs. TT: OR = 0.538, 95% CI = 0.322–0.899, *p* = 0.018; CC + CT vs. TT: OR = 0.586, 95% CI = 0.389–0.884, *p* = 0.011, adjusted by smoking status and age), rather than recessive model. At the same time, the chi‐squared test showed that the C allele could reduce the susceptibility of lung cancer compared with the T allele (OR = 0.735, 95% CI = 0.573–0.942, *p =* 0.015). In the age subgroup stratified by average age (i.e. 58 years old), rs1948915 CT was associated with lower susceptibility of lung cancer in Table 6 (CT vs. TT: OR = 0.645, 95% CI = 0.428–0.972, *p* = 0.036; CC + CT vs. TT: OR = 0.660, 95% CI = 0.449–0.971, *p* = 0.035, adjusted by gender and smoking status). Rs6983267 GT was correlated with lower susceptibility of lung cancer when people ≤58 (GT vs. TT: OR = 0.505, 95% CI = 0.301–0.849, *p* = 0.01; GG vs. GT + TT: OR = 0.561, 95% CI = 0.349–0.903, *p* = 0.017, adjusted by smoking status and gender). Additionally, in the nonsmoking female group of rs1948915, the cases with the CC or CT genotypes could decrease the susceptibility of lung cancer, compared with the homozygous TT (CT vs. TT: OR = 0.624, 95% CI = 0.398–0.980, *p* = 0.040; CC vs. TT: OR = 0.551, 95% CI = 0.325–0.936, *p* = 0.027; CC + CT vs. TT: OR = 0.599, 95% CI = 0.392–0.917, *p* = 0.018, adjusted by age), as shown in Table 5. Using T allele as reference, carriers with C allele had a reduced susceptibility to lung cancer (OR = 0.739, 95% CI = 0.568–0.961, *p* = 0.024).

**TABLE 2:** Association of the selected SNPs with lung cancer and NSCLC

SNP	Control (%)	Lung cancer			NSCLC		
		Case (%)	OR^a^ (95% CI)	*p*	Case (%)	OR^a^ (95% CI)	*p*
rs1948915							
TT	170 (24.4)	191 (28.6)	1 (REF)		149 (27.5)	1 (REF)	
CT	362 (51.9)	325 (48.6)	0.782 (0.600–1.02)	0.069	260 (48.1)	0.806 (0.607–1.068)	0.134
CC	165 (23.7)	153 (22.9)	0.877 (0.641–1.199)	0.410	132 (24.4)	0.981 (0.705–1.365)	0.910
CC + CT vs. TT	527 vs. 170	478 vs. 191	0.811 (0.632–1.041)	0.101	392 vs. 149	0.859 (0.658–1.12)	0.262
CC vs. CT + TT	165 vs. 532	153 vs. 516	1.030 (0.794–1.336)	0.826	132 vs. 409	1.131 (0.861–1.486)	0.376
C vs. T	692 vs. 702	631 vs. 707	0.905 (0.779–1.052)	0.195	524 vs. 558	0.953 (0.813–1.117)	0.549
rs7013433							
TT	203 (29.1)	214 (32.0)	1 (REF)		169 (31.2)	1 (REF)	
AT	346 (49.6)	318 (47.5)	0.887 (0.688–1.143)	0.354	255 (47.1)	0.907 (0.692–1.189)	0.481
AA	148 (21.2)	137 (20.5)	0.961 (0.702–1.314)	0.802	117 (21.6)	1.054 (0.758–1.466)	0.755
AA + AT vs. TT	494 vs. 203	455 vs. 214	0.908 (0.715–1.154)	0.430	372 vs. 169	0.950 (0.737–1.224)	0.689
AA vs. AT + TT	148 vs. 549	137 vs. 532	1.034 (0.789–1.357)	0.806	117 vs. 424	1.119 (0.842–1.488)	0.438
A vs. T	642 vs. 752	592 vs. 746	0.930 (0.799–1.081)	0.342	489 vs. 593	0.966 (0.824–1.133)	0.670
rs6983267							
TT	240 (34.4)	231 (34.5)	1 (REF)		175 (32.3)	1 (REF)	
GT	345 (49.5)	337 (50.4)	0.972 (0.761–1.240)	0.817	277 (51.2)	1.056 (0.814–1.37)	0.681
GG	112 (16.1)	101 (15.1)	0.973 (0.695–1.362)	0.872	89 (16.5)	1.103 (0.775–1.57)	0.585
GG + GT vs. TT	457 vs. 240	438 vs. 231	0.972 (0.771–1.225)	0.809	366 vs. 175	1.067 (0.833–1.367)	0.606
GG vs. GT + TT	112 vs. 585	101 vs. 568	0.989 (0.730–1.341)	0.945	89 vs. 452	1.068 (0.779–1.464)	0.685
G vs. T	569 vs. 825	539 vs. 799	0.978 (0.840–1.140)	0.776	455 vs. 627	1.052 (0.896–1.236)	0.536

Abbreviations: CI, confident interval; OR, odds ratio; REF, reference; SNP, single‐nucleotide polymorphism.

^a^ OR: adjusted by age, gender, and smoking status.

**TABLE 3:** Association of the selected SNPs with lung adenocarcinoma and lung squamous cell carcinoma

SNP	Control (%)	Lung adenocarcinoma			Lung squamous cell carcinoma		
		Case (%)	OR^a^ (95% CI)	*p*	Case (%)	OR^a^ (95% CI)	*p*
rs1948915							
TT	170 (24.4)	85 (30.1)	1 (REF)		62 (25.0)	1 (REF)	
CT	362 (51.9)	130 (46.1)	0.688 (0.491–0.963)	**0.029**	126 (50.8)	1.040 (0.710–1.523)	0.841
CC	165 (23.7)	67 (23.8)	0.828 (0.559–1.229)	0.349	60 (24.2)	1.231 (0.787–1.927)	0.363
CC + CT vs. TT	527 vs. 170	197 vs. 85	0.731 (0.553–1.001)	0.051	186 vs. 62	1.096 (0.763–1.572)	0.620
CC vs. CT + TT	165 vs. 532	67 vs. 215	1.054 (0.755–1.470)	0.758	60 vs. 188	1.199 (0.832–1.729)	0.331
C vs. T	692 vs. 702	264 vs. 300	0.893 (0.734–1.086)	0.256	246 vs. 250	0.998 (0.813–1.225)	0.986
rs7013433							
TT	203 (29.1)	95 (33.7)	1 (REF)		71 (28.6)	1 (REF)	
AT	346 (49.6)	130 (46.1)	0.808 (0.585–1.116)	0.196	121 (48.8)	1.101 (0.763–1.588)	0.608
AA	148 (21.2)	57 (20.2)	0.860 (0.576–1.282)	0.458	56 (22.6)	1.406 (0.901–2.194)	0.133
AA + AT vs. TT	494 vs. 203	187 vs. 95	0.823 (0.608–1.114)	0.207	177 vs. 71	1.183 (0.837–1.671)	0.341
AA vs. AT + TT	148 vs. 549	57 vs. 225	0.977 (0.688–1.388)	0.899	56 vs. 192	1.323 (0.906–1.932)	0.147
A vs. T	642 vs. 752	244 vs. 320	0.893 (0.733–1.088)	0.261	233 vs. 263	1.038 (0.845–1.274)	0.724
rs6983267							
TT	240 (34.4)	92 (32.6)	1 (REF)		81 (32.7)	1 (REF)	
GT	345 (49.5)	149 (52.8)	1.100 (0.803–1.506)	0.554	122 (49.2)	1.004 (0.708–1.423)	0.983
GG	112 (16.1)	41 (14.5)	0.997 (0.642–1.548)	0.990	45 (18.1)	1.220 (0.770–1.932)	0.397
GG + GT vs. TT	457 vs. 240	190 vs. 92	1.075 (0.796–1.452)	0.635	167 vs. 81	1.055 (0.758–1.467)	0.751
GG vs. GT + TT	112 vs. 585	41 vs. 241	0.941 (0.634–1.398)	0.765	45 vs. 203	1.217 (0.808–1.833)	0.348
G vs. T	569 vs. 825	231 vs. 333	1.006 (0.824–1.227)	0.955	212 vs. 284	1.082 (0.880–1.332)	0.455

Abbreviations: CI, confident interval; OR, odds ratio; REF, reference; SNP, single nucleotide polymorphism.

^a^ OR: adjusted by age, gender, and smoking status.

**TABLE 4:** Association between the selected SNPs and lung cancer, stratified by gender

SNP	Gender	Genotype	Case (%)	Control (%)	OR^a^ (95% CI)	*p*
rs1948915	Male	TT	121 (27.7)	113 (26.8)	1 (REF)	
		CT	210 (48.1)	216 (51.2)	0.955 (0.678–1.343)	0.79
		CC	106 (24.3)	93 (22.0)	1.275 (0.849–1.916)	0.242
		CC + CT vs. TT	316 vs. 121	309 vs. 113	1.046 (0.758–1.443)	0.786
		CC vs. CT + TT	106 vs. 331	93 vs. 329	1.314 (0.934–1.849)	0.117
		C vs. T	422 vs. 452	402 vs. 442	1.027 (0.849–1.241)	0.786
	Female	TT	70 (30.2)	57 (20.7)	1 (REF)	
		CT	115 (49.6)	146 (53.1)	0.610 (0.396–0.941)	**0.025**
		CC	47 (20.3)	72 (26.2)	0.538 (0.322–0.899)	**0.018**
		CC + CT vs. TT	162 vs. 70	218 vs. 57	0.586 (0.389–0.884)	**0.011**
		CC vs. CT + TT	47 vs. 185	72 vs. 203	0.749 (0.490–1.142)	0.179
		C vs. T	209 vs. 255	290 vs. 260	0.735 (0.573–0.942)	**0.015**
rs7013433	Male	TT	138 (31.6)	125 (29.6)	1 (REF)	
		AT	204 (46.7)	216 (51.2)	0.915 (0.658–1.273)	0.598
		AA	95 (21.7)	81 (19.2)	1.292 (0.855–1.952)	0.224
		AA + AT v s. TT	299 vs. 138	297 vs. 125	1.011 (0.741–1.380)	0.945
		AA vs. AT + TT	95 vs. 342	81 vs. 341	1.365 (0.954–1.954)	0.089
		A vs. T	394 vs. 480	378 vs. 466	1.012 (0.837–1.224)	0.903
	Female	TT	76 (32.8)	78 (28.4)	1.000 (REF)	
		AT	114 (49.1)	130 (47.3)	0.913 (0.607–1.375)	0.664
		AA	42 (18.1)	67 (24.4)	0.661 (0.399–1.095)	0.108
		AA + AT vs. TT	156 vs. 76	197 vs. 78	0.828 (0.563–1.216)	0.335
		AA vs. AT + TT	42 vs. 190	67 vs. 208	0.699 (0.451–1.083)	0.109
		A vs. T	198 vs. 266	264 vs. 286	0.806 (0.629–1.034)	0.090
rs6983267	Male	TT	141 (32.3)	146 (34.6)	1 (REF)	
		GT	229 (52.4)	205 (48.6)	1.090 (0.793–1.500)	0.594
		GG	67 (15.3)	71 (16.8)	1.024 (0.662–1.582)	0.916
		GG + GT vs. TT	296 vs. 141	276 vs. 146	1.074 (0.793–1.455)	0.644
		GG vs. GT + TT	67 vs. 370	71 vs. 351	0.972 (0.657–1.436)	0.885
		G vs. T	363 vs. 511	347 vs. 497	1.017 (0.840–1.233)	0.860
	Female	TT	90 (38.8)	94 (34.2)	1.000 (REF)	
		GT	108 (46.6)	140 (50.9)	0.788 (0.535–1.162)	0.230
		GG	34 (14.7)	41 (14.9)	0.881 (0.511–1.518)	0.647
		GG + GT vs. TT	142 vs. 90	181 vs. 94	0.809 (0.560–1.169)	0.259
		GG vs. GT + TT	34 vs. 198	41 vs. 234	1.008 (0.613–1.659)	0.974
		G vs. T	176 vs. 288	222 vs. 328	0.903 (0.701–1.163)	0.429

Abbreviations: CI, confident interval; OR, odds ratio; REF, reference; SNP, single nucleotide polymorphism.

^a^ OR: adjusted by age and smoking status.

**TABLE 5:** Association between the selected SNPs and lung cancer in nonsmoking females

SNP	Genotype	Case (%)	Control (%)	OR^a^ (95% CI)	*p*
rs1948915	TT	61 (31.1)	55 (21.2)	1 (REF)	
	CT	93 (47.4)	135 (52.1)	0.624 (0.398–0.980)	**0.040**
	CC	42 (21.4)	69 (26.6)	0.551 (0.325–0.936)	**0.027**
	CC + CT vs. TT	135 vs. 61	204 vs. 55	0.599 (0.392–0.917)	**0.018**
	CC vs. CT + TT	42 vs. 154	69 vs. 190	0.752 (0.485–1.165)	0.202
	C vs. T	177 vs. 215	273 vs. 245	0.739 (0.568–0.961)	**0.024**
rs7013433	TT	63 (32.1)	73 (28.2)	1 (REF)	
	AT	97 (49.5)	122 (47.1)	0.924 (0.601–1.421)	0.720
	AA	36 (18.4)	64 (24.7)	0.652 (0.384–1.107)	0.113
	AA + AT vs. TT	133 vs. 63	186 vs. 73	0.830 (0.554–1.244)	0.368
	AA vs. AT + TT	36 vs. 160	64 vs. 195	0.684 (0.432–1.083)	0.105
	A vs. T	169 vs. 223	250 vs. 268	0.812 (0.624–1.058)	0.123
rs6983267	TT	77 (39.3)	89 (34.4)	1 (REF)	
	GT	89 (45.4)	131 (50.6)	0.785 (0.552–1.179)	0.243
	GG	30 (15.3)	39 (15.1)	0.889 (0.505–1.564)	0.682
	GG + GT vs. TT	119 vs. 77	170 vs. 89	0.809 (0.550–1.118)	0.279
	GG vs. GT + TT	30 vs. 166	39 vs. 220	1.019 (0.608–1.709)	0.942
	G vs. T	149 vs. 243	209 vs. 309	0.907 (0.693–1.186)	0.475

Abbreviations: CI, confident interval; OR, odds ratio; REF, reference; SNP, single nucleotide polymorphism.

^a^ OR: adjusted by age.

**TABLE 6:** Association between the selected SNPs and lung cancer, stratified by average age

SNP	Year	Genotype	Case (%)	Control (%)	OR^a^ (95% CI)	*p*
rs1948915	>58	TT	109 (28.5)	90 (26.7)	1 (REF)	
		CT	189 (49.3)	175 (51.9)	0.919 (0.642–1.316)	0.645
		CC	85 (22.2)	72 (21.4)	1.092 (0.707–1.687)	0.692
		CC + CT vs. TT	274 vs. 109	247 vs. 90	0.968 (0.689–1.359)	0.851
		CC vs. CT + TT	85 vs. 298	72 vs. 265	1.153 (0.799–1.666)	0.446
		C vs. T	359 vs. 407	319 vs. 355	0.982 (0.798–1.208)	0.861
	≤58	TT	82 (28.7)	80 (22.2)	1 (REF)	
		CT	136 (47.6)	187 (51.9)	0.645 (0.428–0.972)	**0.036**
		CC	68 (23.8)	93 (25.8)	0.691 (0.430–1.110)	0.126
		CC + CT vs. TT	204 vs. 82	280 vs. 80	0.660 (0.449–0.971)	**0.035**
		CC vs. CT + TT	68 vs. 218	93 vs. 267	0.925 (0.628–1.362)	0.692
		C vs. T	272 vs. 300	373 vs. 347	0.843 (0.677–1.051)	**0.129**
rs7013433	>58	TT	122 (31.9)	108 (32.0)	1 (REF)	
		AT	180 (47.0)	166 (49.3)	1.003 (0.709–1.418)	0.987
		AA	81 (21.1)	63 (18.7)	1.286 (0.833–1.985)	0.257
		AA + AT vs. TT	261 vs. 122	229 vs. 108	1.078 (0.779–1.493)	0.649
		AA vs. AT + TT	81 vs. 302	63 vs. 274	1.284 (0.877–1.878)	0.199
		A vs. T	342 vs. 424	292 vs. 382	1.055 (0.857–1.300)	0.614
	≤58	TT	92 (32.2)	95 (26.4)	1.000 (REF)	
		AT	138 (48.3)	180 (50.0)	0.756 (0.512–1.117)	0.160
		AA	56 (19.6)	85 (23.6)	0.71 (0.441–1.143)	0.158
		AA + AT vs. TT	194 vs. 92	265 vs. 95	0.742 (0.513–1.071)	0.111
		AA vs. AT + TT	56 vs. 230	85 vs. 275	0.846 (0.563–1.273)	0.422
		A vs. T	250 vs. 322	350 vs. 370	0.821 (0.658–1.023)	0.079
rs6983267	>58	TT	116 (30.3)	118 (35.0)	1 (REF)	
		GT	200 (52.2)	175 (51.9)	1.130 (0.806–1.585)	0.480
		GG	67 (17.5)	44 (13.1)	1.603 (0.998–2.576)	0.051
		GG + GT vs. TT	267 vs. 116	219 vs. 118	1.223 (0.885–1.689)	0.223
		GG vs. GT + TT	67 vs. 316	44 vs. 293	1.487 (0.971–2.278)	0.068
		G vs. T	334 vs. 432	263 vs. 411	1.208 (0.979–1.491)	0.078
	≤58	TT	115 (40.2)	122 (33.9)	1 (REF)	
		GT	137 (47.9)	170 (47.2)	0.831 (0.576–1.199)	0.323
		GG	34 (11.9)	68 (18.9)	0.505 (0.301–0.849)	**0.010**
		GG + GT vs. TT	171 vs. 115	238 vs. 122	0.737 (0.521–1.043)	0.085
		GG vs. GT + TT	34 vs. 252	68 vs. 292	0.561 (0.349–0.903)	**0.017**
		G vs. T	205 vs. 367	306 vs. 414	0.756 (0.603–0.947)	**0.015**

Abbreviations: CI, confident interval; OR, odds ratio; REF, reference; SNP, single nucleotide polymorphism.

^a^ OR: adjusted by gender and smoking status.

**TABLE 7:** Crossover analysis of interaction between the selected SNP genotypes and smoking exposure in lung cancer and NSCLC

SNP	Smoking	Control (%)	Lung cancer			NSCLC		
			Case (%)	OR^a^ (95% CI)	*p*	Case (%)	OR^a^ (95% CI)	*p*
rs1948915								
CT + CC	No	364 (52.2)	231 (34.5)	1 (REF)		192 (35.5)	1 (REF)	
TT	No	117 (16.8)	91 (13.6)	1.251 (0.904–1.731)	0.177	73 (13.5)	1.2 (0.849–1.698)	0.302
CT + CC	Yes	163 (23.4)	247 (36.9)	2.703 (2.019–3.618)	**<0.001**	200 (37.0)	2.531 (1.862–3.442)	**<0.001**
TT	Yes	53 (7.6)	100 (14.9)	3.262 (2.187–4.867)	**<0.001**	76 (14.0)	2.823 (1.852–4.304)	**<0.001**
rs7013433								
AT + AA	No	342 (49.1)	223 (33.3)	1 (REF)		186 (34.4)	1 (REF)	
TT	No	139 (19.9)	99 (14.8)	1.089 (0.797–1.488)	0.594	79 (14.6)	1.036 (0.741–1.447)	0.837
AT + AA	Yes	152 (21.8)	232 (34.7)	2.647 (1.963–3.569)	**<0.001**	186 (34.4)	2.448 (1.787–3.354)	**<0.001**
TT	Yes	64 (9.2)	115 (17.2)	2.961 (2.032–4.312)	**<0.001**	90 (16.6)	2.638 (1.776–3.92)	**<0.001**
rs6983267								
GT + GG	No	313 (44.9)	209 (31.2)	1 (REF)		175 (32.3)	1 (REF)	
TT	No	168 (24.1)	113 (16.9)	1.015 (0.751–1.371)	0.922	90 (16.6)	0.963 (0.698–1.328)	0.818
GT + GG	Yes	144 (20.7)	229 (34.2)	2.643 (1.951–3.581)	**<0.001**	191 (35.3)	2.534 (1.843–3.483)	**<0.001**
TT	Yes	72 (10.3)	118 (17.6)	2.774 (1.921–4.006)	**<0.001**	85 (15.7)	2.282 (1.541–3.379)	**<0.001**

Abbreviations: CI, confident interval; OR, odds ratio; REF, reference; SNP, single nucleotide polymorphism.

^a^ OR: adjusted by age, gender.

**TABLE 8:** Additive interaction between the selected SNP risk genotypes and smoking exposure in lung cancer and NSCLC

SNP	Measure	Lung cancer		NSCLC	
		Estimate	95% CI	Estimate	95% CI
rs1948915	RERI	0.309	−0.971–1.588	0.092	−1.110–1.293
	AP	0.095	−0.272–0.461	0.032	−0.384–0.449
	S	1.158	0.639–2.097	1.053	0.538–2.061
rs7013433	RERI	0.225	−0.887–1.336	0.154	−0.907–1.216
	AP	0.076	−0.282–0.434	0.058	−0.33–0.447
	S	1.130	0.622–2.051	1.104	0.561–2.173
rs6983267	RERI	0.116	−0.918–1.149	−0.215	−1.190–0.760
	AP	0.042	−0.322–0.406	−0.094	−0.54–0.352
	S	1.07	0.586–1.952	0.857	0.423–1.734

Abbreviations: AP, attributable proportion due to interaction; CI, confidence interval; RERI, relative excess risk due to interaction; S, synergy index.

### 3.4 Interaction between the selected SNPs and smoking exposure

It was provided that the results of the crossover analysis are in Table 7. Here, we evaluated the interaction between the selected SNP genotypes and smoking status on lung and NSCLC. We found that smokers with both protective and dangerous genotypes had significantly raised the susceptibility of lung cancer and NSCLC, compared with nonsmokers, indicating that there might be gene–environment interaction. Therefore, we further investigate the interaction, using additive and multiplicative models. Regrettably, there was no interaction of the selected SNP genotypes and smoking exposure with lung cancer and NSCLC risk in both additive and multiplicative models, summed up in Table 8.

## 4 DISCUSSION

It is well known that cancer is a major killer of human health, and lung cancer is the main killer among all cancers. With the present background that growing incidence and bad prognosis of lung cancer have been arousing our great attention, a hospital‐based case–control study was conducted to assess the association between polymorphisms of the three selected SNPs and lung cancer susceptibility. Based on the importance of gene–environment interaction in the development of lung cancer, we further assessed whether there was an interaction between gene polymorphisms and smoking exposure at the selected loci by crossover analysis of SNPs and smoking status.

Through analysis of the GEPIA2 and TCAG databases, we found that the *CCAT1* expression of LUSC tissues was significantly higher than that in normal tissues, while not LUAD tissues. In overall survival time analysis, the Kaplan–Meier curve showed positive results between clinical samples and the expression level of lncRNA *CCAT1* in LUAD patients, but there was no statistical significance of expression level in LUSC patients. A recent study indicated high expression of lncRNA *CCAT1* in NSCLC was correlated with tumor malignant possibility. And lncRNA *CCAT1* directly inhibited microRNA‐218 (miR‐218) and indirectly increased BMI‐1 expression (B lymphoma Mo‐MLV insertion region 1 homolog), then enhanced tumor growth in NSCLC.^38^ The study of ZHAO et al. did not divide NSCLC into subtypes (LUAD and LUSC), which may be the reason of inconsistent results. Herein further validating researches need to be implemented in large and independent samples before a believable conclusion can be drawn.

We obtained that rs1948915 CT/CC significantly decreased the risk of lung cancer in female stratification and even nonsmoking female population, compared with TT genotype carriers. Moreover, compared with their reference genotypes, the results showed that rs1948915 CT and rs6983267 GG had a lower risk for lung cancer in the population ≤ 58 years old. In our result, rs1948915 C and rs6983267 G are protective alleles. However, there was no association of polymorphisms of the three selected SNPs with lung cancer in both overall population and other stratification analyses, including additive and multiplicative models.

Thomsen et al. analyzed European patients with multiple myeloma by GWAS and found that lncRNA *CCAT1* rs1948915 CC genotype was closely related to multiple myeloma,^30^ indicating that allele C was a risk allele. However, in our present study, the results revealed that rs1948915 CT/CC polymorphisms significantly decreased the susceptibility of lung cancer in female population, compared with TT genotype. Obviously, the C allele was protective in lung cancer, with contrast to multiple myeloma. A possible reason is that the expression mechanisms of rs1948915 C/T in *CCAT1* may be different in two cancers, as sophisticated expression mechanisms of rs1948915 polymorphism during the development of two cancers is still unclear.

In the study of rs6983267 on lung cancer, Park et al. found that rs6983267 GG was closely correlated with the susceptibility of lung cancer in smoking stratification through a multicenter case–control study.^33^ However, Zhang et al. performed a case–control study in Zhejiang and Fujian provinces, China, and revealed that individuals carrying the GG homozygous genotype augmented the susceptibility of developing lung cancer compared with ones with TT homozygous genotype. Honestly, in our study, there was no correlation between rs6983267 polymorphism and lung cancer in both overall and stratified population except people no more than 58 years old in age stratification analysis. Moreover, existing evidence has proved that SNP rs6983267 GG could augment the risk of many cancers (e.g. colorectal cancer,^39^, ^40^ gastric cancer,^41^ thyroid cancer,^42^ etc.). The above inconsistence reveals that ethnic or regional differences may be a possible cause, and the other possible reason is the small‐scale sample of our study, which might lead to various deviations. In our stratification analysis, especially in age stratification, the number of subjects in both case group and control group is small, which could result in a false positive. Further mechanism needs to be validated above inconsistent results.

Consequently, the key characteristics of SNPs in carcinogenic lncRNAs are needed to be explored in future study, to discover the unseen capacities of lncRNAs to diagnose early and prevent cancer. In this study, we have clear criteria of inclusion and exclusion in selecting newly diagnosed patients with lung cancer, which can avoid Neyman bias effectively. During the statistical analysis, all the ORs and 95% CIs were adjusted by gender, age, and smoking status in unconditional logistic regression analysis to reduce confounding bias. Nevertheless, we also have some limitations that should not be ignored. First, although we selected cases and controls from multiple hospitals, it is possible to have Berkson bias in the present study. Second, participants in the control were from the physical examination of the same hospital, but it may not represent all the healthy population. Third, the size of our present study is restricted, especially in stratification subgroup. Therefore, large‐scale sample studies are needed to verify the results of our study across different ethnicities and regions later.

## 5 CONCLUSIONS

There may be an association between lung adenocarcinoma and rs1948915 polymorphism in the Chinese northeast population, while rs7013433 and rs6983267 genetic variants might have no association with lung cancer. There was no interaction between the three selected SNPs and smoking status in lung cancer.

## AUTHOR CONTRIBUTIONS

Yangtao Ji conducted experiments and wrote the paper; Yue Yang collected samples and collated data; Zhihua Yin revised the paper. All authors have reviewed the final version of the manuscript and approved to submit to your journal.

## FUNDING INFORMATION

This research was funded by the National Natural Science Foundation of China, grant number 81673261.

## CONFLICT OF INTEREST

The authors declare that they have no conflict of interest.

## Supporting information


Supplementary TableS1

## Data Availability

Data sharing is not applicable to this article as no new data were created or analyzed in this study.

## References

[1] Bray F , Ferlay J , Soerjomataram I , Siegel RL , Torre LA , Jemal A . Global cancer statistics 2018: GLOBOCAN estimates of incidence and mortality worldwide for 36 cancers in 185 countries. CA Cancer J Clin. 2018;68(6):394‐424. 30207593 10.3322/caac.21492

[2] Bray F , Jemal A , Grey N , Ferlay J , Forman D . Global cancer transitions according to the Human Development Index (2008‐2030): a population‐based study. Lancet Oncol. 2012;13(8):790‐801. 22658655 10.1016/S1470-2045(12)70211-5

[3] Siegel RL , Miller KD , Jemal A . Cancer statistics, 2019. CA Cancer J Clin. 2019;69(1):7‐34. 30620402 10.3322/caac.21551

[4] Yin Z , Cui Z , li H , et al. Polymorphisms in miR‐135a‐2, miR‐219‐2 and miR‐211 as well as their interaction with cooking oil fume exposure on the risk of lung cancer in Chinese nonsmoking females: a case‐control study. BMC Cancer. 2016;16(1):751. 27663200 10.1186/s12885-016-2784-1 PMC5035461

[5] Yin Z , Cui Z , Ren Y , et al. Association between polymorphisms in pre‐miRNA genes and risk of lung cancer in a Chinese non‐smoking female population. Lung Cancer (Amsterdam, Netherlands). 2016;94:15‐21. 26973201 10.1016/j.lungcan.2016.01.013

[6] Zhu H , Lv Z , An C , et al. Onco‐lncRNA HOTAIR and its functional genetic variants in papillary thyroid carcinoma. Sci Rep. 2016;6:31969. 27549736 10.1038/srep31969 PMC4994070

[7] Ørom UA , Derrien T , Beringer M , et al. Long noncoding RNAs with enhancer‐like function in human cells. Cell. 2010;143(1):46‐58. 20887892 10.1016/j.cell.2010.09.001 PMC4108080

[8] Li J , Xuan Z , Liu C . Long non‐coding RNAs and complex human diseases. Int J Mol Sci. 2013;14(9):18790‐18808. 24036441 10.3390/ijms140918790 PMC3794807

[9] Huarte M . The emerging role of lncRNAs in cancer. Nat Med. 2015;21(11):1253‐1261. 26540387 10.1038/nm.3981

[10] Zhang A , Xu M , Mo Y‐Y . Role of the lncRNA‐p53 regulatory network in cancer. J Mol Cell Biol. 2014;6(3):181‐191. 24721780 10.1093/jmcb/mju013 PMC4034727

[11] Tao H , Yang JJ , Zhou X , Deng ZY , Shi KH , Li J . Emerging role of long noncoding RNAs in lung cancer: current status and future prospects. Respir Med. 2016;110:12‐19. 26603340 10.1016/j.rmed.2015.10.006

[12] Tao R , Hu S , Wang S , et al. Association between indel polymorphism in the promoter region of lncRNA GAS5 and the risk of hepatocellular carcinoma. Carcinogenesis. 2015;36(10):1136‐1143. 26163879 10.1093/carcin/bgv099

[13] Li L et al. Association between polymorphisms in long non‐coding RNA PRNCR1 in 8q24 and risk of colorectal cancer. J Exper Clin Cancer Res CR. 2013;32:104. 24330491 10.1186/1756-9966-32-104 PMC4029281

[14] Xue Y , Gu D , Ma G , et al. Genetic variants in lncRNA HOTAIR are associated with risk of colorectal cancer. Mutagenesis. 2015;30(2):303‐310. 25432874 10.1093/mutage/geu076

[15] Lv X , Cui Z , Li H , et al. Polymorphism in lncRNA AC008392.1 and its interaction with smoking on the risk of lung cancer in a Chinese population. Cancer Manag Res. 2018;10:1377‐1387. 29881308 10.2147/CMAR.S160818 PMC5985799

[16] Gao M , Li H , Lv X , Zhou B , Yin Z . Association between four polymorphisms in lncRNA and risk of lung cancer in a Chinese never‐smoking female population. DNA Cell Biol. 2018;37(8):651‐658. 29878850 10.1089/dna.2018.4200

[17] Xiang J‐F , Yin QF , Chen T , et al. Human colorectal cancer‐specific CCAT1‐L lncRNA regulates long‐range chromatin interactions at the MYC locus. Cell Res. 2014;24(5):513‐531. 24662484 10.1038/cr.2014.35 PMC4011346

[18] Alaiyan B , Ilyayev N , Stojadinovic A , et al. Differential expression of colon cancer associated transcript1 (CCAT1) along the colonic adenoma‐carcinoma sequence. BMC Cancer. 2013;13:196. 23594791 10.1186/1471-2407-13-196 PMC3639026

[19] Nissan A , Stojadinovic A , Mitrani‐Rosenbaum S , et al. Colon cancer associated transcript‐1: a novel RNA expressed in malignant and pre‐malignant human tissues. Int J Cancer. 2012;130(7):1598‐1606. 21547902 10.1002/ijc.26170

[20] Yang F , Xue X , Bi J , et al. Long noncoding RNA CCAT1, which could be activated by c‐Myc, promotes the progression of gastric carcinoma. J Cancer Res Clin Oncol. 2013;139(3):437‐445. 23143645 10.1007/s00432-012-1324-x PMC11824540

[21] Zhu H‐Q , Zhou X , Chang H , et al. Aberrant expression of CCAT1 regulated by c‐Myc predicts the prognosis of hepatocellular carcinoma. Asian Pac J Cancer Prev. 2015;16(13):5181‐5185. 26225650 10.7314/apjcp.2015.16.13.5181

[22] Guo J , Ma Y , Peng X , Jin H , Liu J . LncRNA CCAT1 promotes autophagy via regulating ATG7 by sponging miR‐181 in hepatocellular carcinoma. J Cell Biochem. 2019;120(10):17975‐17983. 10.1002/jcb.29064 31218739

[23] Zhou B , Wang Y , Jiang J , et al. The long noncoding RNA colon cancer‐associated transcript‐1/miR‐490 axis regulates gastric cancer cell migration by targeting hnRNPA1. IUBMB Life. 2016;68(3):201‐210. 26825578 10.1002/iub.1474

[24] Kim T , Cui R , Jeon YJ , et al. Long‐range interaction and correlation between MYC enhancer and oncogenic long noncoding RNA CARLo‐5. Proc Natl Acad Sci USA. 2014;111(11):4173‐4178. 24594601 10.1073/pnas.1400350111 PMC3964128

[25] Chung CC , Hsing AW , Yeboah E , et al. A comprehensive resequence‐analysis of 250 kb region of 8q24.21 in men of African ancestry. Prostate. 2014;74(6):579‐589. 24783269 10.1002/pros.22726 PMC4199861

[26] He X , Tan X , Wang X , et al. C‐Myc‐activated long noncoding RNA CCAT1 promotes colon cancer cell proliferation and invasion. Tumour Biol. 2014;35(12):12181‐12188. 25185650 10.1007/s13277-014-2526-4

[27] McCleland ML , Mesh K , Lorenzana E , et al. CCAT1 is an enhancer‐templated RNA that predicts BET sensitivity in colorectal cancer. J Clin Invest. 2016;126(2):639‐652. 26752646 10.1172/JCI83265 PMC4731162

[28] Younger ST , Rinn JL . 'Lnc'‐ing enhancers to MYC regulation. Cell Res. 2014;24(6):643‐644. 24777251 10.1038/cr.2014.54 PMC4042177

[29] Zhao X , Wei X , Zhao L , et al. The rs6983267 SNP and long non‐coding RNA CARLo‐5 are associated with endometrial carcinoma. Environ Mol Mutagen. 2016;57(7):508‐515. 27432114 10.1002/em.22031

[30] Thomsen H , Campo C , Weinhold N , et al. Genomewide association study on monoclonal gammopathy of unknown significance (MGUS). Eur J Haematol. 2017;99(1):70‐79. 28375557 10.1111/ejh.12892

[31] Mitchell JS , Li N , Weinhold N , et al. Genome‐wide association study identifies multiple susceptibility loci for multiple myeloma. Nat Commun. 2016;7:12050. 27363682 10.1038/ncomms12050 PMC4932178

[32] Li Y , Jing F , Ding Y , He Q , Zhong Y , Fan C . Long noncoding RNA CCAT1 polymorphisms are associated with the risk of colorectal cancer. Cancer Genet. 2018;222‐223:13‐19. 10.1016/j.cancergen.2018.02.003 29666003

[33] Park SL , Chang SC , Cai L , et al. Associations between variants of the 8q24 chromosome and nine smoking‐related cancer sites. Cancer Epidem Biomark Prevent. 2008;17(11):3193‐3202. 10.1158/1055-9965.EPI-08-0523 PMC2664075 18990762

[34] Zhang X , Chen Q , He C , et al. Polymorphisms on 8q24 are associated with lung cancer risk and survival in Han Chinese. PLoS One. 2012;7(7):e41930. 22848662 10.1371/journal.pone.0041930 PMC3407045

[35] Thean LF , Blöcker C , Li HH , et al. Enhancer‐derived long non‐coding RNAs CCAT1 and CCAT2 at rs6983267 has limited predictability for early stage colorectal carcinoma metastasis. Sci Rep. 2021;11(1):404. 33432117 10.1038/s41598-020-79906-7 PMC7801656

[36] Ozawa T , Matsuyama T , Toiyama Y , et al. CCAT1 and CCAT2 long noncoding RNAs, located within the 8q.24.21 'gene desert', serve as important prognostic biomarkers in colorectal cancer. Ann Oncol. 2017;28(8):1882‐1888. 28838211 10.1093/annonc/mdx248 PMC5834045

[37] Tang Z , Kang B , Li C , Chen T , Zhang Z . GEPIA2: an enhanced web server for large‐scale expression profiling and interactive analysis. Nucl Acids Res. 2019;47(W1):W556‐W560. 31114875 10.1093/nar/gkz430 PMC6602440

[38] Zhao L , Wang L , Wang Y , Ma P . Long non‐coding RNA CCAT1 enhances human non‐small cell lung cancer growth through downregulation of microRNA‐218. Oncol Rep. 2020;43(4):1045‐1052. 32323859 10.3892/or.2020.7500 PMC7057767

[39] Nan H , Morikawa T , Suuriniemi M , et al. Aspirin use, 8q24 single nucleotide polymorphism rs6983267, and colorectal cancer according to CTNNB1 alterations. J Natl Cancer Inst. 2013;105(24):1852‐1861. 24317174 10.1093/jnci/djt331 PMC3866156

[40] Yang B , Thyagarajan B , Gross MD , Goodman M , Sun YV , Bostick RM . Genetic variants at chromosome 8q24, colorectal epithelial cell proliferation, and risk for incident, sporadic colorectal adenomas. Mol Carcinog. 2014;53(Suppl 1):E187‐E192. 23776012 10.1002/mc.22047

[41] Zhou CP , Pan HZ , Li FX , Hu NY , Li M , Yang XX . Association analysis of colorectal cancer susceptibility variants with gastric cancer in a Chinese Han population. Genet Mol Res. 2014;13(2):3673‐3680. 24854447 10.4238/2014.May.9.10

[42] Sahasrabudhe R , Estrada A , Lott P , et al. The 8q24 rs6983267G variant is associated with increased thyroid cancer risk. Endocr Relat Cancer. 2015;22(5):841‐849. 26290501 10.1530/ERC-15-0081 PMC4558310

