# Supplementary material for: Polymorphisms in lncRNA CCAT1 on the susceptibility of lung cancer in a Chinese northeast population: A case–control study
Source: Cancer Med. 2022 Jun 1;12(1):500–12. doi: 10.1002/cam4.4902 (PMC9844612; doi:10.1002/cam4.4902)
Supplement: Supplementary file 1 — Supplementary TableS1 [file CAM4-12-500-s001.docx]

**Supplementary table Association between the selected SNPs and lung cancer, stratified by smoking status**

| **SNP** | **Smoking status** | **Genotype** | **Case (%)** | **Control (%)** | **OR ^a^ (95% CI)** | ***P*** |
| --- | --- | --- | --- | --- | --- | --- |
| rs1948915 | Yes | TT | 100(28.8) | 53(24.5) | 1 (REF) |  |
|  |  | CT | 172(49.6) | 122(56.5) | 0.779(0.514-1.179) | 0.238 |
|  |  | CC | 75(21.6) | 41(19.0) | 1.058(0.631-1.775) | 0.831 |
|  |  | CC + CT vs. TT | 247 vs.100 | 163 vs.53 | 0.848(0.571-1.260) | 0.415 |
|  |  | CC vs. CT + TT | 75 vs.272 | 41 vs.175 | 1.250(0.808-1.932) | 0.316 |
|  |  | C vs. T | 322 vs.372 | 204 vs.228 | 0.967(0.760-1.231) | 0.787 |
|  | No | TT | 91 (28.3) | 117 (24.3) | 1 (REF) |  |
|  |  | CT | 153 (47.5) | 240 (49.9) | 0.803 (0.569-1.133) | 0.211 |
|  |  | CC | 78 (24.2) | 124 (25.8) | 0.804 (0.54-1.197) | 0.282 |
|  |  | CC + CT vs. TT | 231 vs.91 | 364 vs.117 | 0.803 (0.581-1.109) | 0.183 |
|  |  | CC vs. CT + TT | 78 vs.244 | 124 vs.357 | 0.927 (0.667-1.29) | 0.654 |
|  |  | C vs. T | 309 vs.335 | 488 vs.474 | 0.896 (0.734-1.094) | 0.281 |
| rs7013433 | Yes | TT | 115 (33.1) | 64 (29.6) | 1 (REF) |  |
|  |  | AT | 166 (47.9) | 117 (54.2) | 0.856 (0.576-1.271) | 0.440 |
|  |  | AA | 66 (19.0) | 35 (16.2) | 1.12 (0.664-1.888) | 0.671 |
|  |  | AA + AT vs. TT | 232 vs.115 | 152 vs.64 | 0.917 (0.629-1.336) | 0.651 |
|  |  | AA vs. AT + TT | 66 vs.281 | 35 vs.181 | 1.234 (0.779-1.955) | 0.370 |
|  |  | A vs. T | 298 vs.396 | 187 vs.245 | 0.986 (0.774-1.257) | 0.909 |
|  | No | TT | 99 (30.7) | 139 (28.9) | 1 (REF) |  |
|  |  | AT | 152 (47.2) | 229 (47.6) | 0.930(0.667-1.296) | 0.667 |
|  |  | AA | 71 (22.1) | 113 (23.5) | 0.896 (0.602-1.332) | 0.586 |
|  |  | AA + AT vs. TT | 223 vs.99 | 342 vs.139 | 0.918 (0.673-1.253) | 0.592 |
|  |  | AA vs. AT + TT | 71 vs.251 | 113 vs.368 | 0.937 (0.666-1.317) | 0.707 |
|  |  | A vs. T | 294 vs.350 | 455 vs.507 | 0.936 (0.766-1.143) | 0.517 |
| rs6983267 | Yes | TT | 118(34.0) | 72(33.3) | 1 (REF) |  |
|  |  | GT | 188(54.2) | 109(50.5) | 1.025(0.698-1.506) | 0.898 |
|  |  | GG | 41(11.8) | 35(16.2) | 0.679(0.391-1.178) | 0.168 |
|  |  | GG + GT vs. TT | 229 vs. 118 | 144 vs. 72 | 0.941(0.652-1.359) | 0.746 |
|  |  | GG vs. GT + TT | 41 vs. 306 | 35 vs. 181 | 0.669(0.406-1.101) | 0.114 |
|  |  | G vs. T |  |  | 0.900(0.705-1.150) | 0.399 |
|  | No | TT | 113(35.1) | 168(34.9) | 1 (REF) |  |
|  |  | GT | 149(46.3) | 236(49.1) | 0.930(0.676-1.278) | 0.653 |
|  |  | GG | 60(18.6) | 77(16.0) | 1.179(0.776-1.791) | 0.441 |
|  |  | GG + GT vs. TT | 209 vs.113 | 313 vs.168 | 1.010(0.749-1.363) | 0.947 |
|  |  | GG vs. GT + TT | 60 vs.262 | 77 vs.404 | 1.229(0.844-1.790) | 0.282 |
|  |  | G vs. T | 269 vs.375 | 390 vs.572 | 1.052(0.859-1.289) | 0.623 |

SNP: single nucleotide polymorphism; OR: odds ratio; CI: confident interval; REF: reference

OR ^a^: adjusted by gender and age
